# Association of New Use of Antihypertensives That Stimulate vs Inhibit Type 2 and 4 Angiotensin II Receptors With Dementia Among Medicare Beneficiaries

**DOI:** 10.1001/jamanetworkopen.2022.49370

**Published:** 2023-01-04

**Authors:** Zachary A. Marcum, Nico Gabriel, Adam P. Bress, Inmaculada Hernandez

**Affiliations:** 1 Department of Pharmacy, University of Washington School of Pharmacy, Seattle; 2 Division of Clinical Pharmacy, Skaggs School of Pharmacy and Pharmaceutical Sciences, University of California, San Diego, La Jolla; 3 Division of Health System Innovation and Research, Department of Population Health Sciences, University of Utah School of Medicine, Salt Lake City; 4 George E. Wahlen Department of Veterans Affairs Medical Center, Salt Lake City, Utah; 5 Department of Internal Medicine, University of Utah School of Medicine, Salt Lake City

## Abstract

**Importance:**

Prevalent use of antihypertensive medications that stimulate type 2 and 4 angiotensin II receptors, compared with those that do not stimulate these receptors, has been associated with a lower risk of dementia. However, previous studies were limited by inclusion of individuals with prevalent hypertension and a history of antihypertensive use prior to the start of the study, which can introduce bias.

**Objective:**

To examine the association of new use of antihypertensive medication regimens that stimulate vs inhibit type 2 and 4 angiotensin II receptors with Alzheimer disease and related dementias (ADRD) among Medicare beneficiaries.

**Design, Setting, and Participants:**

This cohort study was conducted among 57 773 Medicare fee-for-service beneficiaries (January 1, 2006, through December 31, 2018) aged 65 years or older with incident hypertension. Data analysis was conducted from January 1 through June 30, 2022.

**Exposures:**

Initiation of antihypertensive medication regimens that stimulate or inhibit type 2 and 4 angiotensin II receptors, or mixed regimens (both stimulating and inhibiting), with the time-dependent measure being each 30-day interval.

**Main Outcomes and Measures:**

The primary outcome was time to first occurrence of ADRD (Centers for Medicare & Medicaid Services Chronic Conditions Data Warehouse definition). Cox proportional hazards regression modeling with time-dependent variables was performed to estimate the association between time-dependent treatment groups and time to ADRD, after adjusting for sociodemographic and clinical characteristics.

**Results:**

The sample included 57 773 Medicare beneficiaries (36 348 women [62.9%]; mean [SD] age, 73.8 [6.3] years; 2954 [5.1%] Black, 1545 [2.7%] Hispanic; 50 184 [86.9%] White, and 3090 [5.4%] Other individuals [the Other category included individuals of American Indian, Asian, other, or unknown race and ethnicity]). During a median of 6.9 years (IQR, 4.7-9.3 years) of follow-up, the unadjusted incidence density rate of ADRD was 2.2 cases per 100 person-years (95% CI, 2.1-2.4 cases per 100 person-years) for the group receiving regimens that stimulate type 2 and 4 angiotensin II receptors compared with 3.1 cases per 100 person-years (95% CI, 3.0-3.2 cases per 100 person-years) for the group receiving regimens that inhibit type 2 and 4 angiotensin II receptors and 2.7 cases per 100 person-years (95% CI, 2.6-2.9 cases per 100 person-years) for the group receiving mixed treatment regimens. In adjusted Cox proportional hazards regression modeling, stimulating treatment was associated with a statistically significant 16% reduction in the hazard of ADRD compared with inhibiting treatment (hazard ratio, 0.84; 95% CI, 0.79-0.90). Mixed regimen use was also associated with reduced hazards of ADRD compared with the inhibiting group (hazard ratio, 0.90; 95% CI, 0.84-0.96).

**Conclusions and Relevance:**

This cohort study of Medicare beneficiaries suggests that use of antihypertensive medications that stimulate type 2 and 4 angiotensin II receptors was associated with lower risk of ADRD compared with antihypertensive medications that inhibit these receptors. Confirmation is needed in a randomized trial.

## Key Points

**Question:**

Is initiation of antihypertensive medications that stimulate vs inhibit type 2 and 4 angiotensin II receptors associated with a lower risk of dementia?

**Findings:**

In this cohort study of 57 773 Medicare beneficiaries, initiation of antihypertensive medications that stimulate vs inhibit type 2 and 4 angiotensin II receptors was associated with a statistically significant 16% lower risk of incident dementia, over a median of 6.9 years of follow-up.

**Meaning:**

The findings of this study suggest that initiation of antihypertensive medications that stimulate vs inhibit type 2 and 4 angiotensin II receptors may result in a lower risk of incident dementia; confirmation is needed in a randomized clinical trial.

## Teaser

This cohort study compares the risk of incident Alzheimer disease and related dementias between treatment regimens with antihypertensive medications that stimulate type 2 and 4 angiotensin II receptors compared with those that inhibit type 2 and 4 angiotensin II receptors among Medicare beneficiaries with hypertension.

## Introduction

Hypertension is one of the leading modifiable risk factors for Alzheimer disease and related dementias (ADRD).^1,2^ Mechanistic, observational, and randomized clinical studies suggest that certain classes of antihypertensive medication may reduce cognitive impairment independent of their blood pressure–lowering effects.^3,4,5,6,7^ Randomized clinical trials provide the highest-quality evidence for estimating causal effects. However, randomized clinical trials testing interventions for dementia prevention (eg, antihypertensive medication repurposing) are cost-prohibitive and time-prohibitive due to the long latency period of cognitive decline. Generating high-quality and actionable inferences by using modern data analysis techniques is important to complement and extend trial findings and inform clinical and policy decisions regarding ADRD prevention in a timely and cost-efficient manner.

Previous observational studies have reported that prevalent use of regimens containing antihypertensive medications that stimulate type 2 and 4 angiotensin II receptors, compared with those that do not, were associated with lower rates of incident mild cognitive impairment and dementia.^8,9^ However, those studies were limited by the inclusion of individuals with prevalent hypertension and relatively small sample sizes. Using an active-comparator, new-user design and a nationally representative cohort study of Medicare beneficiaries, we sought to compare the risk of incident ADRD between treatment regimens with antihypertensive medications that stimulate type 2 and 4 angiotensin II receptors (angiotensin II receptor type 1 blockers, dihydropyridine calcium channel blockers, thiazide diuretics) and regimens with antihypertensive medications that inhibit type 2 and 4 angiotensin II receptors (angiotensin-converting enzyme inhibitors, β-blockers, nondihydropyridine calcium channel blockers).

## Methods

### Study Design

**Figure 1.**
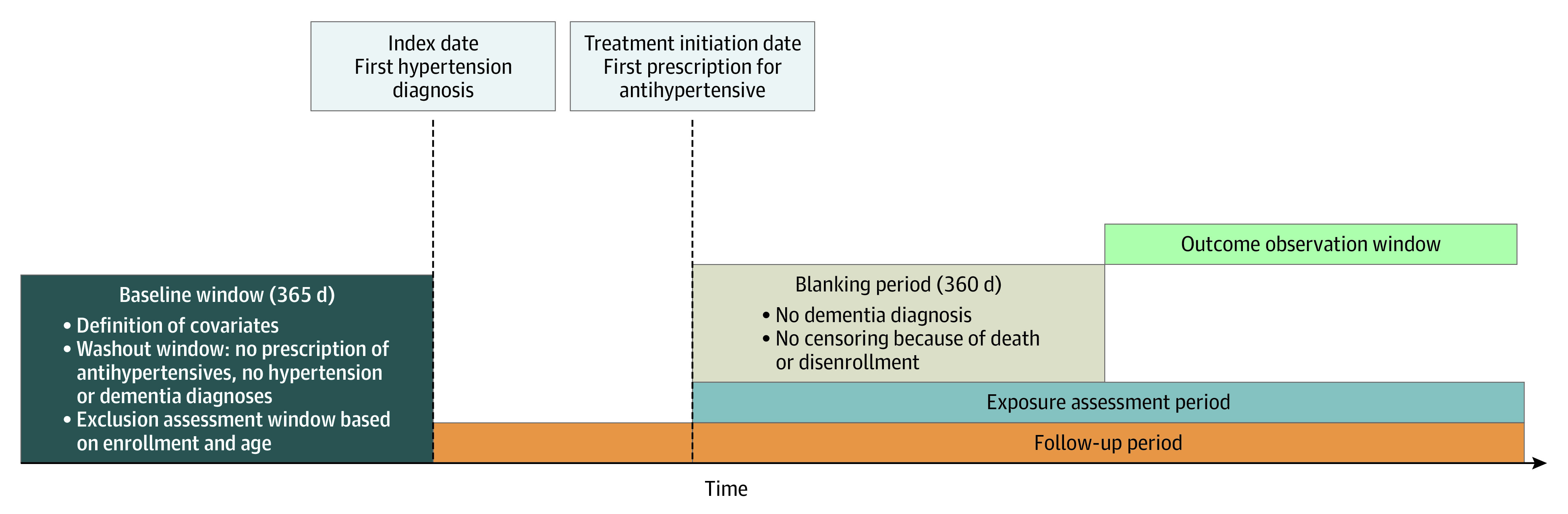
Study Design This is a new-user study design, with the exposure of interest being antihypertensive medication use after the first hypertension diagnosis (index date). Patients were included in the study if they had a new diagnosis of hypertension between January 1, 2007, and December 31, 2014. To ensure patients had a new diagnosis of hypertension, a 365-day baseline washout period prior to the index date was applied. Use of antihypertensive medication was ascertained starting on the index date. The day of the first fill of an antihypertensive medication after the index date was defined as the treatment initiation date. Outcome events were measured starting 360 days after the treatment initiation date because Alzheimer disease and related dementia and vascular dementia develop over time; therefore, a minimum exposure was assumed to be needed before collecting outcome events. Outcome events were assessed from the end of the blanking period to death, disenrollment, or end of the study period (December 31, 2018).

We conducted a retrospective cohort study of Medicare beneficiaries with hypertension. We used Medicare claims data from January 1, 2006, through December 31, 2018, for a 5% random sample of Medicare beneficiaries from the Centers for Medicare & Medicaid Services. Figure 1 shows the study design, outlining the timing of measurement of antihypertensive medication use and study outcomes. Beneficiaries were included in the study if they had a new diagnosis of hypertension from January 1, 2007, to December 31, 2014; the first occurrence of a diagnosis was defined as the index date. This time frame allowed for at least 4 years of follow-up for all study participants (because data were available through 2018). To ensure beneficiaries had a new diagnosis of hypertension, we applied a 365-day baseline washout period prior to the index date. Beneficiaries with a diagnosis of hypertension or a claim for an antihypertensive medication during this 365-day washout period were excluded. We ascertained use of antihypertensive medication starting on the index date. The day of the first fill for an antihypertensive medication that stimulates or inhibits type 2 and 4 angiotensin II receptors (list of medications in eTable 1 in Supplement 1) after the index date was defined as the treatment initiation date. We measured outcome events (ADRD and vascular dementia) starting 360 days after the treatment initiation date. We applied this blanking period because ADRD and vascular dementia develop over time; therefore, we assumed a minimum exposure was needed for the antihypertensive medication to plausibly have an appreciable association with cognitive outcomes. Outcome events were collected from the end of the blanking period until the occurrence of an outcome event, death, disenrollment, or end of the study period. This study was deemed exempt by the institutional review board at the University of California, San Diego, because it used deidentified claims data. Informed consent was not obtained due to the exempt status of this study. This report follows the Strengthening the Reporting of Observational Studies in Epidemiology (STROBE) reporting guideline for cohort studies.

### Data Source and Study Participants

We used claims data from January 1, 2006, through December 31, 2018, for a 5% random sample of Medicare beneficiaries and selected the sample in 8 steps (eFigure 1 in Supplement 1). First, we identified beneficiaries with an inpatient or outpatient claim for hypertension from January 1, 2007, to December 31, 2014, using *International Classification of Diseases, Ninth Revision* (*ICD-9*) and *International Statistical Classification of Diseases and Related Health Problems, Tenth Revision* (*ICD-10*) codes listed in eTable 2 in Supplement 1. Second, to ensure the sample was representative of beneficiaries with a new diagnosis of hypertension, we required continuous enrollment in the Medicare fee-for-service program (Part A, Part B, and a stand-alone prescription drug plan) in the 12 months prior to the first diagnosis of hypertension. Third, we excluded beneficiaries with a diagnosis of hypertension in this baseline period, as they could have represented prevalent cases of hypertension. Fourth, we constrained sampling to beneficiaries who filled at least 1 pharmacy claim for an angiotensin II receptor type 2 and 4–stimulating or –inhibiting antihypertensive medication after the index date. Fifth, we excluded beneficiaries who filled a prescription for these medications in the baseline period, as they would not be new users. Sixth, we excluded beneficiaries who developed ADRD or vascular dementia before filling a pharmacy claim for an angiotensin II receptor type 2 and 4–stimulating or –inhibiting antihypertensive medication. Seventh, we excluded beneficiaries who died, developed ADRD or vascular dementia, or disenrolled from the Medicare fee-for-service program by the end of the 360-day blanking period. Eighth, we constrained sampling to beneficiaries older than 65 years as of the index date.

### Main Exposure

To define the exposures of interest, we extracted all prescriptions for antihypertensive medications starting on the index date. Angiotensin II receptor type 2 and 4–stimulating antihypertensive medications (hereafter, *stimulating medications*) included angiotensin II receptor type 1 blockers, dihydropyridine calcium channel blockers, and thiazide diuretics. Angiotensin II receptor type 2 and 4–inhibiting antihypertensive medications (hereafter, *inhibiting medications*) included angiotensin-converting enzyme inhibitors, β-blockers, and nondihydropyridine calcium channel blockers. Using the prescription fill date and the days of supply, we created a supply diary. The supply diary assessed whether beneficiaries had possession of stimulating medication and inhibiting medication each day of the exposure assessment window. Each 30-day interval, we categorized beneficiaries into 4 time-dependent treatment groups: (1) the stimulating medication group, defined as possessing stimulating medication 80% or more of the time (≥24 days of the 30-day interval) and possessing inhibiting medication less than 80% of the time (<24 days of the 30-day interval); (2) the inhibiting medication group, defined as possessing inhibiting medication 80% or more of the time (≥24 days of the 30-day interval) and possessing stimulating medication less than 80% of the time (<24 days of the 30-day interval); (3) the mixed group, defined as possessing inhibiting medication 80% or more of the time (≥24 days of the 30-day interval) and possessing stimulating medication 80% or more of the time (≥24 days of the 30-day interval); and (4) the nonusers group, defined as possessing inhibiting medication less than 80% of the time (<24 days of the 30-day interval) and possessing stimulating medication less than 80% of the time (<24 days of the 30-day interval). We followed this method, previously used in the literature,^10^ because 80% of time covered is the most commonly used threshold to define medication adherence. In a sensitivity analysis, we relaxed the definition of medication possession and defined use of a treatment group when patients had at least 1 day of the 30-day interval covered with medication (ie, >0% days covered).

### Outcomes

The primary outcome was time to first occurrence of ADRD and the secondary outcome was time to first occurrence of vascular dementia. For ADRD, we used the Centers for Medicare & Medicaid Services Chronic Conditions Data Warehouse definition, which defines ADRD has having 1 inpatient or outpatient claim with the *ICD-9* or *ICD-10* codes (eTable 3 in Supplement 1).^11^ The specificity of ADRD using Medicare claims has been estimated to be 89%.^12^ Compared with data from the Consortium to Establish a Registry for Alzheimer Disease, the sensitivity of ADRD using Medicare claims was estimated to be 87% for beneficiaries continuously enrolled in the Medicare fee-for-service program, which is consistent with our analytic sample.^13^ A similar estimate for the sensitivity of Medicare claims for measuring ADRD (87%) was also reported against a criterion standard clinical diagnosis of dementia measured in the University of Southern California Alzheimer Disease Research Center.^14^ Vascular dementia was defined as having an inpatient or outpatient claim with *ICD-9* codes 290.40, 290.41, 290.42, and 290.43 or *ICD-10* code F01.

### Covariates

Covariates were selected a priori based on their potential role as confounders (ie, variables that are associated with antihypertensive medication use and cognitive outcomes) (Table 1). Sociodemographic characteristics were coded as time-fixed variables and included age, sex, race and ethnicity, and receipt of low-income subsidy. Race and ethnicity were measured from Medicare’s enrollment database and assessed in this study as potential confounders. The racial and ethnic categories included Black, Hispanic, White, or Other race and ethnicity; Other included American Indian, Asian, other, or unknown race and ethnicity. Comorbidities were coded as time-dependent variables and included time since first hypertension diagnosis, as well as indicator variables for a diagnosis of atrial fibrillation, ischemic heart disease, diabetes, chronic heart failure, depression, chronic kidney disease, and stroke or transient ischemic attack. For additional medication use variables, we included time-fixed indicator variables for anticholinergic medication use^15^ (eTable 4 in Supplement 1), nonsteroidal anti-inflammatory drug use, and statin use, all defined in the 12 months before the index date, and a count variable for other antihypertensive (eg, loop diuretic) use in the 12 months before the index date.

**Table 1.** Baseline Characteristics of Patients by Treatment Group Defined on the First 30-Day Interval After Index Date^a^

Sociodemographic characteristic	Medication type, No. (%)				
	Type 2 and 4 angiotensin II–stimulating (n = 4879)	Type 2 and 4 angiotensin II–inhibiting (n = 10 303)	Mixed (n = 2179)	Nonusers (n = 40 413)	Total (N = 57 773)
Age, (SD), y	73.6 (6.43)	73.7 (6.33)	73.1 (5.97)	73.9 (6.31)	73.8 (6.3)
Sex					
Female	3261 (66.8)	6263 (60.8)	1337 (61.4)	25 487 (63.1)	36 348 (62.9)
Male	1612 (33.0)	4039 (39.2)	842 (38.6)	14 926 (36.9)	21 419 (37.1)
Race and ethnicity					
Black	403 (8.3)	382 (3.7)	226 (10.4)	1943 (4.8)	2954 (5.1)
Hispanic	166 (3.4)	331 (3.2)	95 (4.4)	953 (2.4)	1545 (2.7)
White	3959 (81.1)	9068 (88.0)	1730 (79.4)	35 428 (87.7)	50 184 (86.9)
Other^b^	351 (7.2)	522 (5.1)	128 (5.9)	2089 (5.2)	3090 (5.4)
Low-income subsidy	1534 (31.4)	2987 (29.0)	808 (37.1)	10 017 (24.8)	15 346 (26.6)
Prior medical history					
Atrial fibrillation	88 (1.8)	416 (4.0)	33 (1.5)	1820 (4.5)	2357 (4.1)
Chronic heart failure	276 (5.7)	962 (9.3)	152 (7.0)	4225 (10.5)	5615 (9.7)
Chronic kidney disease	217 (4.4)	409 (4.0)	74 (3.4)	2316 (5.7)	3016 (5.2)
Depression	732 (15.0)	1619 (15.7)	255 (11.7)	7246 (17.9)	9852 (17.1)
Diabetes	422 (8.6)	1631 (15.8)	218 (10.0)	7497 (18.6)	9768 (16.9)
Ischemic heart disease	818 (16.8)	2817 (27.3)	371 (17.0)	11 709 (29.0)	15 715 (27.2)
Stroke	208 (4.3)	576 (5.6)	92 (4.2)	2432 (6.0)	3308 (5.7)
Prior medication history					
Anticholinergics^c^	852 (17.5)	1826 (17.7)	288 (13.2)	7756 (19.2)	10 722 (18.6)
NSAIDs	960 (19.7)	1913 (18.6)	349 (16.0)	7873 (19.5)	11 095 (19.2)
Statin	1084 (22.2)	2548 (24.7)	352 (16.2)	10 987 (27.2)	14 971 (25.9)
Other antihypertensives, No. (SD)^d^	0.06 (0.25)	0.08 (0.28)	0.06 (0.25)	0.13 (0.38)	0.11 (0.35)

Abbreviation: NSAIDs, nonsteroidal anti-inflammatory drugs.

^a^ The table compares baseline characteristics across treatment groups, defined based on treatment groups on the first 30-day interval after index date. Type 2 and 4 angiotensin II receptor–stimulating antihypertensive medications were defined as angiotensin II receptor blockers, dihydropyridine calcium channel blockers, and/or thiazides. Type 2 and 4 angiotensin II receptor–inhibiting antihypertensive medications were defined as angiotensin-converting enzyme inhibitors, β-blockers, and/or nondihydropyridine calcium channel blockers. Mixed antihypertensive medications were defined as both type 2 and 4 angiotensin II receptor–stimulating and –inhibiting antihypertensive medications. For all comparisons across treatment groups, *P* < .001 by analysis of variance (continuous variables) or χ^2^ test (categorical variables).

^b^ Defined as Asian, American Indian, other, or unknown from the Centers for Medicare & Medicaid Services.

^c^ Anticholinergics defined via the Anticholinergic Cognitive Burden Score (values of 2 or 3).

^d^ Defined as antihypertensive drugs other than those included in the definition of type 2 and 4 angiotensin II receptor–stimulating and –inhibiting antihypertensive medications.

### Statistical Analysis

Statistical analysis was conducted from January 1 to June 30, 2022. We compared patient characteristics across treatment groups defined by the first 30-day period after index date using analysis of variance for continuous variables and the χ^2^ test for categorical variables. We calculated the incidence of outcomes in each time-dependent treatment group per 100 person-years and reported cumulative incidences at equally distributed time points over follow-up (years 3, 6, and 9, and study end). We constructed Cox proportional hazards regression models with time-dependent variables to compare the incidence of outcomes across treatment groups, while adjusting for the covariates listed above. Time zero was the end of the blanking period (360 days after treatment initiation date), and the time at risk was censored at death, disenrollment from the Medicare fee-for-service program, or the end of the study period (December 31, 2018). The inhibiting medication group was selected as the reference group for treatment comparisons. Covariate variables were lagged to the 30-day interval prior to ensure that the exposure was measured prior to the measure of the outcome. In a sensitivity analysis, we relaxed the exposure variable definition from medication possession for at least 24 days of the 30-day interval (≥80% days covered) to at least 1 day of the 30-day interval (ie, >0% days covered). All analyses were performed with SAS statistical software, version 9.4 (SAS Institute Inc). All *P* values were from 2-sided tests and results were deemed statistically significant at *P* < .05.

## Results

### Beneficiary Characteristics by First 30-Day Interval After Index Date

The final sample included 57 773 Medicare beneficiaries (36 348 [62.9%] were women and 21 419 [37.1%] were men; mean [SD] age, 73.8 [6.3] years; 2954 [5.1%] Black, 1545 [2.7%] Hispanic; 50 184 [86.9%] White, and 3090 [5.4%] Other individuals [the Other category included individuals of American Indian, Asian, other, or unknown race and ethnicity]). Table 1 compares beneficiary characteristics across time-fixed treatment groups, defined according to medication use in the first 30-day interval after the index date. Age was similar across all groups. In the first 30-day interval, new users of stimulating compared with inhibiting regimens had a higher proportion of women (66.8% vs 60.8%) and Black beneficiaries (8.3% vs 3.7%). New users of stimulating (vs inhibiting) regimens also had a lower prevalence of atrial fibrillation (1.8% vs 4.0%), chronic heart failure (5.7% vs 9.3%), diabetes (8.6% vs 15.8%), and ischemic heart disease (16.8% vs 27.3%). New users of stimulating (vs inhibiting) regimens had similar use of anticholinergics (17.5% vs 17.7%), nonsteroidal anti-inflammatory drugs (19.7% vs 18.6%), and statins (22.2% vs 24.7%) prior to the index date.

### Mean Follow-up Time in Time-Dependent Treatment Groups

**Figure 2.**
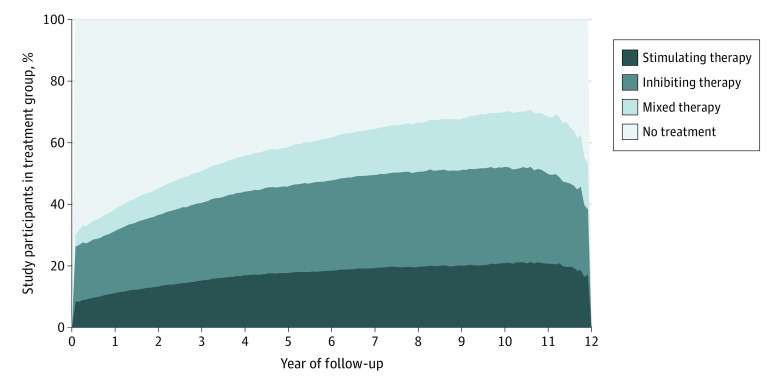
Proportion of Patients in Each Time-Dependent Exposure Group During Follow-up Treatment group was defined in a time-dependent fashion, at each 30-day interval. Type 2 and 4 angiotensin II receptor–stimulating antihypertensive medications were defined as angiotensin II receptor blockers, dihydropyridine calcium channel blockers, and/or thiazides. Type 2 and 4 angiotensin II receptor–inhibiting antihypertensive medications were defined as angiotensin-converting enzyme inhibitors, β-blockers, and/or nondihydropyridine calcium channel blockers. Mixed antihypertensive medications were defined as both type 2 and 4 angiotensin II receptor–stimulating and –inhibiting antihypertensive medications.

Beneficiaries spent 67 118 person-years (15.5% of follow-up time) in the stimulating treatment group, 112 076 person-years (26.4% of follow-up time) in the inhibiting treatment group, 48 997 person-years (10.9% of follow-up time) in the mixed treatment group, and 199 902 person-years (47.2% of follow-up time) in the nonuser group (Figure 2). eFigure 2 and eTable 6 in Supplement 1 depict transitions across time-dependent treatment groups over study follow-up.

### Primary Outcome

Over a median of 6.9 years (IQR, 4.7-9.3 years) of follow-up, the unadjusted incidence density rate of ADRD was 2.2 cases per 100 person-years (95% CI, 2.1-2.4 cases per 100 person-years) for the stimulating medication group (1425 total events) compared with 3.1 cases per 100 person-years (95% CI, 3.0-3.2 cases per 100 person-years) for the inhibiting medication group (3266 total events) and 2.7 cases per 100 person-years (95% CI, 2.6-2.9 cases per 100 person-years) for the mixed group (1244 total events) (Table 2).

**Table 2.** Follow-up and Incidence Density Rates of the Primary Outcome, by Time-Dependent Treatment Group^a^

Follow-up	Medication type			
	Type 2 and 4 angiotensin II–stimulating medications	Type 2 and 4 angiotensin II–inhibiting medications	Mixed	Nonusers
Year 3				
Patient-years, No.	20 302	36 318	13 092	97 875
No. of patients with a new diagnosis of ADRD	223	617	184	962
Cumulative incidence (95% CI), per 100 person-years	1.1 (1.0-1.3)	1.7 (1.6-1.8)	1.4 (1.2-1.6)	1.0 (0.9-1.0)
Year 6				
Patient-years, No.	43 017	72 775	29 138	155 096
No. of patients with a new diagnosis of ADRD	728	1741	600	2693
Cumulative incidence (95% CI), per 100 person-years	1.7 (1.6-1.8)	2.4 (2.3-2.5)	2.1 (1.9-2.2)	1.7 (1.7-1.8)
Year 9				
Patient-years, No.	58 227	96 560	41 097	182 411
No. of patients with a new diagnosis of ADRD	1191	2836	1029	4059
Cumulative incidence (95% CI), per 100 person-years	2.0 (1.9-2.2)	2.9 (2.8-3.0)	2.5 (2.4-2.7)	2.2 (2.2-2.3)
Study end				
Patient-years, No.	63 662	104 657	45 815	190 605
No. of patients with a new diagnosis of ADRD	1425	3266	1244	4653
Cumulative incidence (95% CI), per 100 person-years	2.2 (2.1-2.4)	3.1 (3.0-3.2)	2.7 (2.6-2.9)	2.4 (2.4-2.5)

Abbreviation: ADRD, Alzheimer disease and related dementias.

^a^ Data in this table were binned into larger categories of time increments for table presentation. To do so, we summed the information for all 30-day intervals in the first 3 years of the study, at year 6, at year 9, and at study end. Treatment group was defined in a time-dependent fashion, at each 30-day interval. Type 2 and 4 angiotensin II receptor–stimulating antihypertensive medications were defined as angiotensin II receptor blockers, dihydropyridine calcium channel blockers, and/or thiazides. Type 2 and 4 angiotensin II receptor–inhibiting antihypertensive medications were defined as angiotensin-converting enzyme inhibitors, β-blockers, and/or nondihydropyridine calcium channel blockers. Mixed antihypertensive medications were defined as both type 2 and 4 angiotensin II receptor–stimulating and –inhibiting antihypertensive medications.

**Figure 3.**
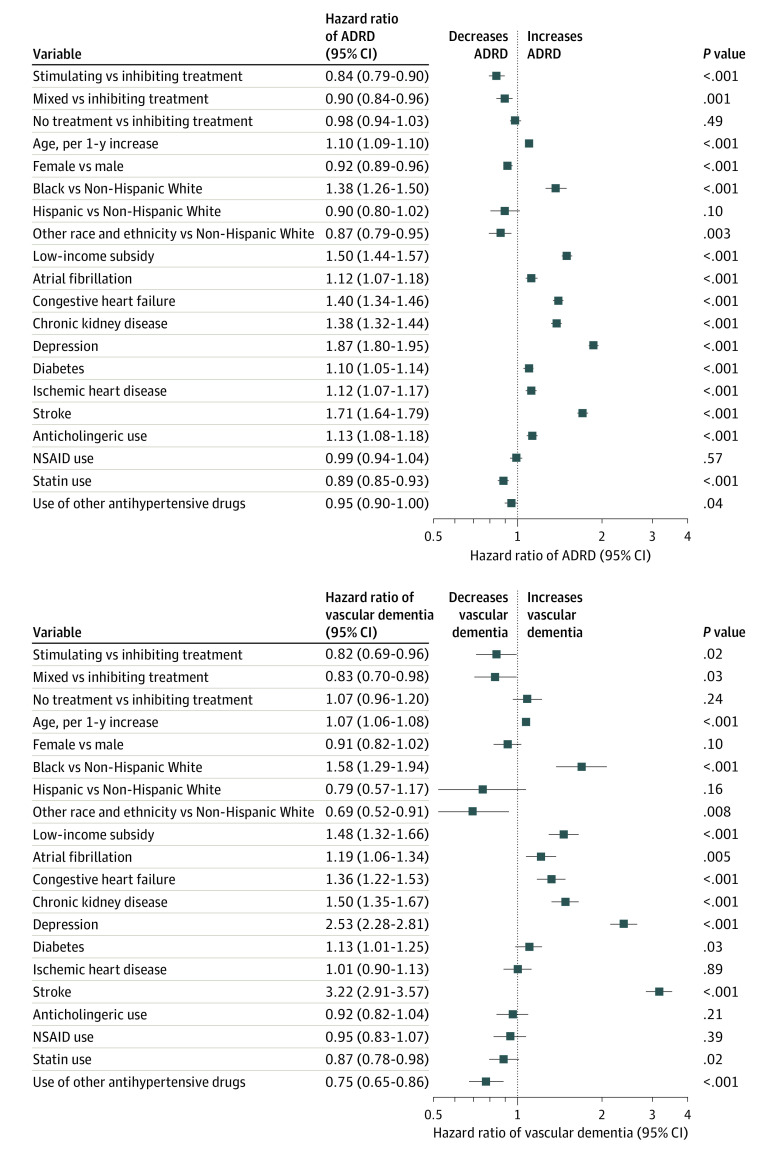
Multivariable Cox Proportional Hazards Regression Model for Primary (Alzheimer Disease and Related Dementia [ADRD]) and Secondary (Vascular Dementia) Outcomes Type 2 and 4 angiotensin II receptor–stimulating antihypertensive medications were defined as angiotensin II receptor blockers, dihydropyridine calcium channel blockers, and/or thiazides. Type 2 and 4 angiotensin II receptor–inhibiting antihypertensives were defined as angiotensin-converting enzyme inhibitors, β-blockers, and/or nondihydropyridine calcium channel blockers. Mixed antihypertensive medications were defined as both type 2 and 4 angiotensin II receptor–stimulating and –inhibiting antihypertensive medications. NSAID indicates nonsteroidal anti-inflammatory drug.

In adjusted Cox proportional hazards regression models, stimulating medication use was associated with reduced hazards of ADRD compared with the inhibiting medication group (hazard ratio [HR], 0.84; 95% CI, 0.79-0.90; 4691 total events for these 2 treatment groups) (Figure 3). Mixed regimen use was also associated with reduced hazards of ADRD compared with the inhibiting medication group (HR, 0.90; 95% CI, 0.84-0.96; 4510 total events for these 2 treatment groups).

Cox proportional hazards regression models also identified sociodemographic characteristics, comorbidities, and medication classes associated with time to ADRD (Figure 3). For instance, age (HR, 1.10; 95% CI, 1.09-1.10 per 1-year increase) and Black race (HR, 1.38; 95% CI, 1.26-1.50) were associated with increased hazards of ADRD. In addition, diagnoses of depression (HR, 1.87; 95% CI, 1.80-1.95) and stroke (HR, 1.71; 95% CI, 1.64-1.79) were associated with increased hazards of ADRD, as was anticholinergic use (HR, 1.13; 95% CI, 1.08-1.18), among other factors.

### Secondary Outcome

Over a median of 7.4 years (IQR, 4.9-9.7 years) of follow-up, the unadjusted incidence density rate of vascular dementia was 0.28 cases per 100 person-years (95% CI, 0.24-0.32 cases per 100 person-years) for the stimulating medication group compared with 0.46 cases per 100 person-years (95% CI, 0.42-0.50 cases per 100 person-years) for the inhibiting medication group and 0.37 cases per 100 person-years (95% CI, 0.32-0.43 cases per 100 person-years) for the mixed group (eTable 5 in Supplement 1).

In adjusted Cox proportional hazards regression models, stimulating medication use was associated with reduced hazards of vascular dementia compared with the inhibiting group (HR, 0.82; 95% CI, 0.69-0.96; 700 total events for these 2 treatment groups) (Figure 3). Mixed regimen use was also associated with reduced hazards of vascular dementia compared with the inhibiting group (HR, 0.83; 95% CI, 0.70-0.98; 692 total events for these 2 treatment groups).

Sociodemographic characteristics and comorbidities associated with time to vascular dementia were similar to the primary outcome model (Figure 3). Age (HR, 1.07; 95% CI, 1.06-1.08 per 1-year increase) and Black race (HR, 1.58; 95% CI, 1.29-1.94) were associated with increased hazards of vascular dementia. In addition, a diagnosis of depression (HR, 2.53; 95% CI, 2.28-2.81) and stroke (HR, 3.22; 95% CI, 2.91-3.57), among other factors, were associated with increased hazards of vascular dementia.

### Sensitivity Analysis

Results were consistent for our sensitivity analysis when use of a treatment was defined using a 1-day threshold instead of a 24-day threshold (eFigure 3 in Supplement 1).

## Discussion

In this cohort study of Medicare beneficiaries with incident hypertension, initiation of an antihypertensive medication regimen that exclusively stimulates vs inhibits type 2 and 4 angiotensin II receptors was associated with a 16% lower risk (HR, 0.84; 95% CI, 0.79-0.90) of incident ADRD over approximately 7 years of follow-up. Use of stimulating vs inhibiting medications was also associated with a 18% lower risk (HR, 0.82; 95% CI, 0.69-0.96) of vascular dementia. Results were independent of cardiovascular risk factors and sociodemographic characteristics and were consistent with changes in the definition of the exposure.

The results of the current analysis extend prior work testing this hypothesis in the Prevention of Dementia by Intensive Vascular Care (PreDIVA) trial^8^ and the Systolic Blood Pressure Intervention Trial (SPRINT).^9^ Van Dalen et al^8^ found that prevalent users of only stimulating antihypertensive medications had a 45% lower risk of incident dementia compared with users of only inhibiting antihypertensive medications over 6.7 years of follow-up in the PreDIVA trial. In addition, Marcum et al^9^ found that prevalent users of only stimulating antihypertensive medications had a 25% lower risk of incident amnestic mild cognitive impairment or probable dementia compared with users of only inhibiting antihypertensive medications over 4.8 years of follow-up in SPRINT. As opposed to these previous studies, the current study included only beneficiaries with incident hypertension and new use of antihypertensive medications as well as adjustment for time-varying confounding—none of these study design and analysis procedures were used in the PreDIVA trial or SPRINT. Because randomized clinical trials provide the strongest evidence for estimating causal effects of treatments, the current study may warrant confirmation in a clinical trial. Medications that inhibit angiotensin II type 2 and 4 receptors will continue to have an important place in the antihypertensive treatment armamentarium. However, on a population level, shifting prescribing from inhibiting to stimulating regimens could reduce dementia risk.^16^

Animal and human studies support potential underlying mechanisms for these findings. The overarching hypothesis that, beyond their implications for blood pressure, antihypertensive drugs that increase activity at the type 2 and 4 angiotensin II receptors provide greater brain protection compared with those that decrease activity, is supported by a sizable volume of animal model studies^17,18,19,20,21,22,23,24^ and human studies.^25,26,27,28^ Studies suggest a role for angiotensin II and angiotensin IV activity in protection from ischemia or enhanced cerebral blood flow, especially via activity at angiotensin type 2 and, possibly, type 4 receptors.^29,30,31,32^ Agonism at the angiotensin type 4 receptor may improve spatial memory processing.^21,32,33,34^ However, there remains unresolved complexity in understanding the interaction among antihypertensives, the renin–angiotensin system, and cognitive outcomes.

### Limitations

This study has several limitations. First, we included beneficiaries with continuous enrollment in the Medicare fee-for-service program, so our results are not generalizable to Medicare Advantage beneficiaries or populations exposed to antihypertensives at an earlier age. Second, it is known that dementia is underdiagnosed,^35^ leading to likely outcome underascertainment. However, it is unlikely that this outcome measurement error would occur differentially by antihypertensive treatment groups. Third, the most important unmeasured confounder in this analysis is blood pressure due to the nature of claims data. We do not anticipate differential blood pressure–lowering effects of our main treatment comparison (stimulating vs inhibiting), although future studies should examine whether antihypertensive dose is associated with ADRD. Fourth, our time-dependent definition of exposure to medication use was unable to distinguish nonusers as those with previous use of a medication from those without a history of using the medication. Nonusers were included in this analysis because the modeling was performed on a time-dependent basis. However, the results for the nonuser group are not relevant for inference in this comparative effectiveness analysis. In addition, delays in dementia diagnosis can introduce potential bias, but we are unable to assess this using claims data, as we do not have information on the onset of cognitive symptoms. Despite these limitations, we controlled for measured confounding through statistical adjustment of baseline and time-varying, pretreatment covariates and reduced unmeasured confounding through study design parameters by restricting the sample to incident treated hypertension and new users of antihypertensive medications.

## Conclusions

In this cohort study of Medicare beneficiaries with incident hypertension, antihypertensive medications that stimulate vs inhibit type 2 and 4 angiotensin II receptors were associated with lower rates of dementia among new users of these medications. Although we adjusted for a set of baseline and time-varying covariates, as with all observational studies, we cannot rule out the possibility of residual confounding. Confirmation of these findings is needed in a clinical trial.

## Notes

Supplement 1. **eTable 1.** Antihypertensive Medications (Generic Name) by Category **eTable 2.** *ICD-9* and *ICD-10* Diagnosis Codes for Hypertension **eTable 3.** *ICD-9* and *ICD-10* Diagnosis Codes for ADRD **eTable 4.** Medications (Generic Names) in the Anticholinergic Cognitive Burden Score (Values of 2 or 3) **eTable 5.** Follow-up and Incidence Density Rates of the Secondary Outcome, by Time-Dependent Treatment Group **eTable 6.** Individuals in Exposure Groups by Time Period After Index Date, Among Those Remaining in the Study **eFigure 1.** Flowchart of Sample Selection **eFigure 2.** Sankey Diagram of Exposure Groups by Time Period After Index Date **eFigure 3.** Results of Sensitivity Analysis - Multivariable Cox Proportional Hazards Model for Primary (ADRD) and Secondary (Vascular Dementia) Outcomes

Supplement 2.
Data Sharing Statement

## References

[1] Livingston G, Huntley J, Sommerlad A, . Dementia prevention, intervention, and care: 2020 report of the Lancet Commission. Lancet. 2020;396(10248):413-446. doi:10.1016/S0140-6736(20)30367-6 32738937 PMC7392084

[2] National Academies of Sciences, Engineering, and Medicine. *Preventing Cognitive Decline and Dementia: A Way Forward*. National Academies Press; 2017. 28650595

[3] Kehoe PG. The coming of age of the angiotensin hypothesis in Alzheimer’s disease: progress toward disease prevention and treatment? J Alzheimers Dis. 2018;62(3):1443-1466. doi:10.3233/JAD-171119 29562545 PMC5870007

[4] Levi Marpillat N, Macquin-Mavier I, Tropeano A-I, Bachoud-Levi A-C, Maison P. Antihypertensive classes, cognitive decline and incidence of dementia: a network meta-analysis. J Hypertens. 2013;31(6):1073-1082. doi:10.1097/HJH.0b013e3283603f53 23552124

[5] den Brok MGHE, van Dalen JW, Abdulrahman H, . Antihypertensive medication classes and the risk of dementia: a systematic review and network meta-analysis. J Am Med Dir Assoc. 2021;22(7):1386-1395. doi:10.1016/j.jamda.2020.12.019 33460618

[6] Hajjar I, Okafor M, McDaniel D, . Effects of candesartan vs lisinopril on neurocognitive function in older adults with executive mild cognitive impairment: a randomized clinical trial. JAMA Netw Open. 2020;3(8):e2012252. doi:10.1001/jamanetworkopen.2020.12252 32761160 PMC7411539

[7] ClinicalTrials.gov. Risk Reduction for Alzheimer’s Disease (rrAD). ClinicalTrials.gov identifier: NCT02913664. Accessed June 19, 2022. https://clinicaltrials.gov/ct2/show/NCT02913664

[8] van Dalen JW, Marcum ZA, Gray SL, . Association of angiotensin II–stimulating antihypertensive use and dementia risk: post hoc analysis of the PreDIVA trial. Neurology. 2021;96(1):e67-e80. doi:10.1212/WNL.0000000000010996 33154085 PMC7884979

[9] Marcum ZA, Cohen JB, Zhang C, ; Systolic Blood Pressure Intervention Trial (SPRINT) Research Group. Association of antihypertensives that stimulate vs inhibit types 2 and 4 angiotensin II receptors with cognitive impairment. JAMA Netw Open. 2022;5(1):e2145319. doi:10.1001/jamanetworkopen.2021.45319 35089354 PMC8800076

[10] Hernandez I, He M, Brooks MM, Saba S, Gellad WF. Adherence to anticoagulation and risk of stroke among Medicare beneficiaries newly diagnosed with atrial fibrillation. Am J Cardiovasc Drugs. 2020;20(2):199-207. doi:10.1007/s40256-019-00371-3 31523759 PMC7073283

[11] Chronic Conditions Data Warehouse. Data dictionaries. Accessed June 19, 2022. https://www2.ccwdata.org/web/guest/data-dictionaries

[12] Taylor DH Jr, Østbye T, Langa KM, Weir D, Plassman BL. The accuracy of Medicare claims as an epidemiological tool: the case of dementia revisited. J Alzheimers Dis. 2009;17(4):807-815. doi:10.3233/JAD-2009-1099 19542620 PMC3697480

[13] Taylor DH Jr, Fillenbaum GG, Ezell ME. The accuracy of Medicare claims data in identifying Alzheimer’s disease. J Clin Epidemiol. 2002;55(9):929-937. doi:10.1016/S0895-4356(02)00452-3 12393082

[14] Lee E, Gatz M, Tseng C, . Evaluation of Medicare claims data as a tool to identify dementia. J Alzheimers Dis. 2019;67(2):769-778. doi:10.3233/JAD-181005 30689589 PMC7164318

[15] Aging Brain Program, Indiana University Center for Aging Research. Anticholinergic Cognitive Burden Scale: 2012 update. Accessed June 19, 2022. http://www.idhca.org/wp-content/uploads/2018/02/DESAI_ACB_scale_-_Legal_size_paper.pdf

[16] Marcum ZA, Cohen JB, Larson EB, Williamson J, Bress AP. Can preferentially prescribing angiotensin II receptor blockers (ARBs) over angiotensin-converting enzyme inhibitors (ACEIs) decrease dementia risk and improve brain health equity? NAM Perspect. Published online May 9, 2022. doi:10.31478/202205c 36177210 PMC9499377

[17] Faure S, Chapot R, Tallet D, Javellaud J, Achard JM, Oudart N. Cerebroprotective effect of angiotensin IV in experimental ischemic stroke in the rat mediated by AT_4_ receptors. J Physiol Pharmacol. 2006;57(3):329-342. 17033088

[18] Hamai M, Iwai M, Ide A, . Comparison of inhibitory action of candesartan and enalapril on brain ischemia through inhibition of oxidative stress. Neuropharmacology. 2006;51(4):822-828. doi:10.1016/j.neuropharm.2006.05.029 16824557

[19] Iwai M, Inaba S, Tomono Y, . Attenuation of focal brain ischemia by telmisartan, an angiotensin II type 1 receptor blocker, in atherosclerotic apolipoprotein E–deficient mice. Hypertens Res. 2008;31(1):161-168. doi:10.1291/hypres.31.161 18360031

[20] Iwai M, Liu H-W, Chen R, . Possible inhibition of focal cerebral ischemia by angiotensin II type 2 receptor stimulation. Circulation. 2004;110(7):843-848. doi:10.1161/01.CIR.0000138848.58269.80 15289370

[21] Lee J, Albiston AL, Allen AM, . Effect of I.C.V. injection of AT_4_ receptor ligands, NLE^1^-angiotensin IV and LVV-hemorphin 7, on spatial learning in rats. Neuroscience. 2004;124(2):341-349. doi:10.1016/j.neuroscience.2003.12.006 14980384

[22] Li J, Culman J, Hörtnagl H, . Angiotensin AT2 receptor protects against cerebral ischemia–induced neuronal injury. FASEB J. 2005;19(6):617-619. doi:10.1096/fj.04-2960fje 15665034

[23] Lu Q, Zhu YZ, Wong PTH. Neuroprotective effects of candesartan against cerebral ischemia in spontaneously hypertensive rats. Neuroreport. 2005;16(17):1963-1967. doi:10.1097/01.wnr.0000187636.13147.cd 16272888

[24] Wang J, Ho L, Chen L, . Valsartan lowers brain β-amyloid protein levels and improves spatial learning in a mouse model of Alzheimer disease. J Clin Invest. 2007;117(11):3393-3402. doi:10.1172/JCI31547 17965777 PMC2040315

[25] Boutitie F, Oprisiu R, Achard JM, . Does a change in angiotensin II formation caused by antihypertensive drugs affect the risk of stroke? a meta-analysis of trials according to treatment with potentially different effects on angiotensin II. J Hypertens. 2007;25(8):1543-1553. doi:10.1097/HJH.0b013e32814a5ae5 17620946

[26] Epstein BJ, Gums JG. Can the renin-angiotensin system protect against stroke? a focus on angiotensin II receptor blockers. Pharmacotherapy. 2005;25(4):531-539. doi:10.1592/phco.25.4.531.61022 15977915

[27] Hajjar I, Brown L, Mack WJ, Chui H. Impact of angiotensin receptor blockers on Alzheimer disease neuropathology in a large brain autopsy series. Arch Neurol. 2012;69(12):1632-1638. doi:10.1001/archneurol.2012.1010 22964777 PMC3608189

[28] Hajjar I, Levey A. Association between angiotensin receptor blockers and longitudinal decline in tau in mild cognitive impairment. JAMA Neurol. 2015;72(9):1069-1070. doi:10.1001/jamaneurol.2015.1001 26368351 PMC4752362

[29] Saavedra JM, Benicky J, Zhou J. Mechanisms of the anti-ischemic effect of angiotensin II AT_1_ receptor antagonists in the brain. Cell Mol Neurobiol. 2006;26(7-8):1099-1111. doi:10.1007/s10571-006-9009-0 16636899 PMC11520717

[30] Braszko JJ, Walesiuk A, Wielgat P. Cognitive effects attributed to angiotensin II may result from its conversion to angiotensin IV. J Renin Angiotensin Aldosterone Syst. 2006;7(3):168-174. doi:10.3317/jraas.2006.027 17094054

[31] Chai SY, Bastias MA, Clune EF, . Distribution of angiotensin IV binding sites (AT_4_ receptor) in the human forebrain, midbrain and pons as visualised by in vitro receptor autoradiography. J Chem Neuroanat. 2000;20(3-4):339-348. doi:10.1016/S0891-0618(00)00112-5 11207430

[32] Pederson ES, Harding JW, Wright JW. Attenuation of scopolamine-induced spatial learning impairments by an angiotensin IV analog. Regul Pept. 1998;74(2-3):97-103. doi:10.1016/S0167-0115(98)00028-7 9712169

[33] Pederson ES, Krishnan R, Harding JW, Wright JW. A role for the angiotensin AT_4_ receptor subtype in overcoming scopolamine-induced spatial memory deficits. Regul Pept. 2001;102(2-3):147-156. doi:10.1016/S0167-0115(01)00312-3 11730987

[34] Wright JW, Stubley L, Pederson ES, Kramár EA, Hanesworth JM, Harding JW. Contributions of the brain angiotensin IV-AT_4_ receptor subtype system to spatial learning. J Neurosci. 1999;19(10):3952-3961. doi:10.1523/JNEUROSCI.19-10-03952.1999 10234025 PMC6782731

[35] Barnes DE, Zhou J, Walker R, . Development and validation of the Electronic Health Record Risk of Alzheimer’s and Dementia Assessment Rule (eRADAR). J Am Geriatr Soc. 2020;68(1):103-111. doi:10.1111/jgs.16182 31612463 PMC7094818

