# Supplementary material for: Association of New Use of Antihypertensives That Stimulate vs Inhibit Type 2 and 4 Angiotensin II Receptors With Dementia Among Medicare Beneficiaries
Source: JAMA Netw Open. 2023 Jan 4;6(1):e2249370. doi: 10.1001/jamanetworkopen.2022.49370 (PMC9856661; doi:10.1001/jamanetworkopen.2022.49370)
Supplement: Supplement 1. — eTable 1. Antihypertensive Medications (Generic Name) by Category eTable 2. ICD-9 and ICD-10 Diagnosis Codes for Hypertension eTable 3. ICD-9 and ICD-10 Diagnosis Codes for ADRD eTable 4. Medications (Generic Names) in the Anticholinergic Cognitive Burden Score (Values of 2 or 3) eTable 5. Follow-up and Incidence Density Rates of the Secondary Outcome, by Time-Dependent Treatment Group eTable 6. Individuals in Exposure Groups by Time Period After Index Date, Among Those Remaining in the Study eFigure 1. Flowchart of Sample Selection eFigure 2. Sankey Diagram of Exposure Groups by Time Period After Index Date eFigure 3. Results of Sensitivity Analysis - Multivariable Cox Proportional Hazards Model for Primary (ADRD) and Secondary (Vascular Dementia) Outcomes [file jamanetwopen-e2249370-s001.pdf]

Supplementary Online Content

Marcum ZA, Gabriel N, Bress AP, Hernandez I. Association of new use of antihypertensives that stimulate vs inhibit type 2 and 4 angiotensin II receptors with dementia among Medicare beneficiaries. *JAMA Netw Open.* 2023;6(1):e2249370. doi:10.1001/jamanetworkopen.2022.49370

eTable 1. Antihypertensive Medications (Generic Name) by Category
eTable 2. *ICD-9* and *ICD-10* Diagnosis Codes for Hypertension
eTable 3. *ICD-9* and *ICD-10* Diagnosis Codes for ADRD
eTable 4. Medications (Generic Names) in the Anticholinergic Cognitive Burden Score (Values of 2 or 3)
eTable 5. Follow-up and Incidence Density Rates of the Secondary Outcome, by Time-Dependent Treatment Group
eTable 6. Individuals in Exposure Groups by Time Period After Index Date, Among Those Remaining in the Study
eFigure 1. Flowchart of Sample Selection
eFigure 2. Sankey Diagram of Exposure Groups by Time Period After Index Date
eFigure 3. Results of Sensitivity Analysis - Multivariable Cox Proportional Hazards Model for Primary (ADRD) and Secondary (Vascular Dementia) Outcomes

This supplementary material has been provided by the authors to give readers additional information about their work.

© 2023 Marcum ZA et al. *JAMA Network Open.*

eTable 1. Antihypertensive Medications (Generic Name) by Category

**Angiotensin II type 2 and 4 stimulating**	**Angiotensin II type 2 and 4 inhibiting**	**Other**
amlodipine	acebutolol	acetazolamide
azilsartan	atenolol	aliskiren
bendroflumethiazide	benazepril	ambrisentan
candesartan	bepridil	amiloride
chlorothiazide	betaxolol	bosentan
chlorthalidone	bisoprolol	bumetanide
eprosartan	captopril	clonidine
felodipine	carteolol	deserpidine
hydrochlorothiazide	carvedilol	dipyridamole
hydroflumethiazide	diltiazem	doxazosin
indapamide	enalapril	eplerenone
irbesartan	fosinopril	ergoloid mesylates
isradipine	labetalol	ethacrynic acid
losartan	lisinopril	ethaverine
methyclothiazide	metoprolol	furosemide
nicardipine	mibefradil	guanabenz
nifedipine	moexipril	guanadrel
nimodipine	nadolol	guanethidine
nisoldipine	nebivolol	guanfacine
olmesartan	penbutolol	hydralazine
polythiazide	perindopril	isoxsuprine
telmisartan	pindolol	Macitentan
trichlormethiazide	propranolol	mannitol
valsartan	quinapril	mecamylamine
	ramipril	methyldopa
	sotalol	metolazone
	timolol	minoxidil
	trandolapril	pamabrom
	verapamil	papaverine
	verapamil	papaverine
		phenoxybenzamine
		prazosin
		rauwolfia serpentina
		reserpine
		riociguat
		sacubitril
		selexipag
		spironolactone
		terazosin
		tolvaptan
		torsemide
		torsemide
		treprostinil
		triamterene

© 2023 Marcum ZA et al. *JAMA Network Open.*

eTable 2. *ICD-9* and *ICD-10* Diagnosis Codes for Hypertension

**ICD-9**	362.11, 401.0, 401.1, 401.9, 402.00, 402.01, 402.10, 402.11, 402.90, 402.91, 403.00, 403.01, 403.10, 403.11, 403.90, 403.91, 404.00, 404.01, 404.02, 404.03, 404.10, 404.11, 404.12, 404.13, 404.90, 404.91, 404.92, 404.93, 405.01, 405.09, 405.11, 405.19, 405.91, 405.99, 437.2
**ICD-10**	H35.031, H35.032, H35.033, H35.039, I10, I11.0, I11.9, I12.0, I12.9, I13.0, I13.10, I13.11, I13.2, I15.0, I15.1, I15.2, I15.8, I15.9, I67.4, N26.2

© 2023 Marcum ZA et al. *JAMA Network Open.*

eTable 3. *ICD-9* and *ICD-10* Diagnosis Codes for ADRD

**ICD-9 (any position)**	331.0, 331.11, 331.19, 331.2, 331.7, 290.0, 290.10, 290.11, 290.12, 290.13, 290.20, 290.21, 290.3, 290.40, 290.41, 290.42, 290.43, 294.0, 294.10, 294.11, 294.20, 294.21, 294.8, 797
**ICD-10 (any position)**	F01.50, F01.51, F02.80, F02.81, F03.90, F03.91, F04, F05, F06.1, F06.8, G13.8, G30.0, G30.1, G30.8, G30.9, G31.01, G31.09, G31.1, G31.2, G94, R41.81, R54

Source: https://www2.ccwdata.org/web/guest/condition-categories-chronic

© 2023 Marcum ZA et al. *JAMA Network Open.*

eTable 4. Medications (Generic Names) in the Anticholinergic Cognitive Burden Score (Values of 2 or 3)

Anticholinergc Cognitive Burden Scale
**Generic name**
Amitriptyline
Amoxapine
Atropine
Benztropine
Brompheniramine
Carbinoxamine
Chlorpheniramine
Chlorpromazine
Clemastine
Clomipramine
Clozapine
Darifenacin
Desipramine
Dicyclomine
Dimenhydrinate
Diphenhydramine
Doxepin
Doxylamine
Fesoterodine
Flavoxate
Hydroxyzine
Hyoscyamine
Imipramine
Meclizine
Methocarbamol
Nortriptyline
Olanzapine
Orphenadrine
Oxybutynin
Paroxetine
Perphenazine
Promethazine
Propantheline
Propiverine
Quetiapine
Scopolamine
Solifenacin
Thioridazine
Tolterodine
Trifluoperazine
Trihexyphenidyl

© 2023 Marcum ZA et al. *JAMA Network Open.*

Trimipramine
Trospium
Amantadine
Belladonna Multiple
Carbamazepine
Cyclobenzaprine
Cyproheptadine
Loxapine
Meperidine
Methotrimeprazine
Molindone
Nefopam
Oxcarbazepine
Pimozide

© 2023 Marcum ZA et al. *JAMA Network Open.*

eTable 5. Follow-up and Incidence Density Rates of the Secondary Outcome, by Time-Dependent Treatment Group[a]

Follow-up	AT2R/AT4R Stimulating	AT2R/AT4R Inhibiting	Mixed	Non-Users
**At year 3**	**-**	**-**	**-**	**-**
**Patient-years**	20478	36760	13245	98452
**No. patients newly diagnosed with vascular dementia**	14	55	14	86
**Cumulative incidence per 100 person- years (95% CI)**	0.07 (0.04-0.12)	0.15 (0.12-0.20)	0.11 (0.06-0.18)	0.09 (0.07-0.11)
**At year 6**	-	-	-	-
**Patient-years**	44159	75285	30110	158359
**No. patients newly diagnosed with vascular dementia**	79	221	73	368
**Cumulative incidence per 100 person- years (95% CI)**	0.18 (0.14-0.22)	0.29 (0.26-0.34)	0.24 (0.19-0.31)	0.23 (0.21-0.26)
**At year 9**	-	-	-	-
**Patient-years**	60647	101661	43199	188808
**No. patients newly diagnosed with vascular dementia**	146	406	153	602
**Cumulative incidence per 100 person- years (95% CI)**	0.24 (0.21-0.28)	0.40 (0.36-0.44)	0.35 (0.30-0.42)	0.32 (0.29-0.35)
**At study end**	-	-	-	-
**Patient-years**	66796	111155	48603	198640
**No. patients newly diagnosed with vascular dementia**	187	513	179	725
**Cumulative incidence per 100 person- years (95% CI)**	0.28 (0.24-0.32)	0.46 (0.42-0.50)	0.37 (0.32-0.43)	0.37 (0.34-0.39)

Abbreviations: AT2R, angiotensin II receptor type 2; AT4R, angiotensin II receptor type 4; CI, confidence interval

[a]Treatment group was defined in a time dependent fashion, at each 30-day interval. Angiotensin II receptor type 2 and 4-stimulating antihypertensives defined as use of angiotensin II receptor blockers, dihydropyridine calcium channel blockers, and/or thiazides. Angiotensin II receptor type 2 and 4-inhibiting antihypertensives defined as use of angiotensin-converting enzyme inhibitors, ß-blockers, and/or non-dihydropyridine calcium channel blockers. Mixed antihypertensive users defined as both angiotensin II receptor type 2 and 4-stimulating and -inhibiting antihypertensives.

© 2023 Marcum ZA et al. *JAMA Network Open.*

eTable 6. Individuals in Exposure Groups by Time Period After Index Date, Among Those Remaining in the Study

	Year 0	Year 3	Year 6	Year 9
Stimulating, n (% of total)	4,879 (8.4%)	7,917 (15.4%)	6,434 (18.5%)	3,377 (20.1%)
Inhibiting, n (% of total)	10,302 (17.8%)	12,981 (25.3%)	10,220 (29.4%)	5,248 (31.2%)
Mixed, n (% of total)	2,179 (3.8%)	5,382 (10.5%)	4,861 (14.0%)	2,795 (16.6%)
None, n (% of total)	40,413 (70.0%)	24,977 (48.7%)	13,268 (38.1%)	5,407 (32.1%)
Total, n	57,773	51,257	34,783	16,827

© 2023 Marcum ZA et al. *JAMA Network Open.*

eFigure 1. Flowchart of Sample Selection

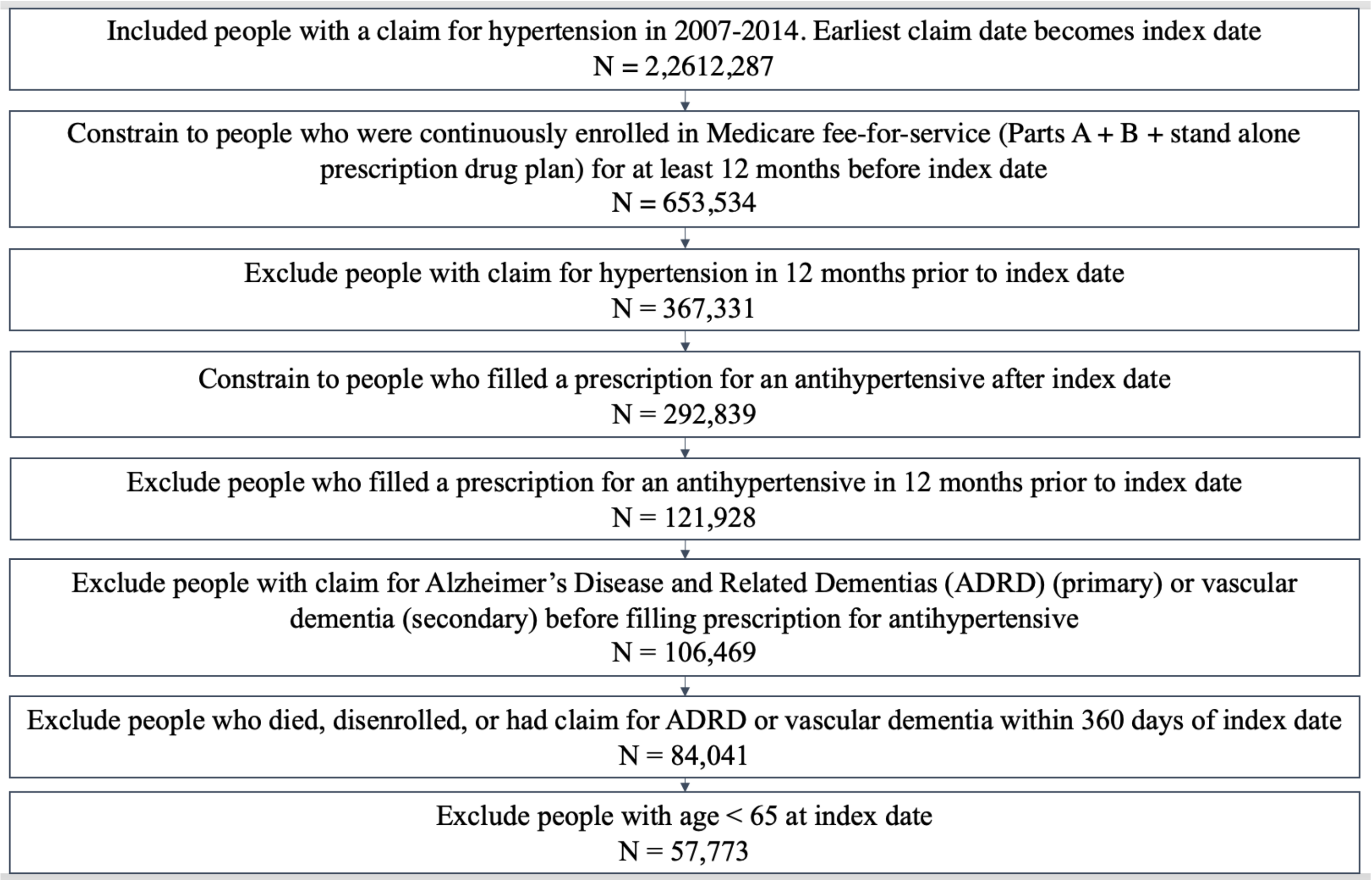

© 2023 Marcum ZA et al. *JAMA Network Open.*

eFigure 2. Sankey Diagram of Exposure Groups by Time Period After Index Date

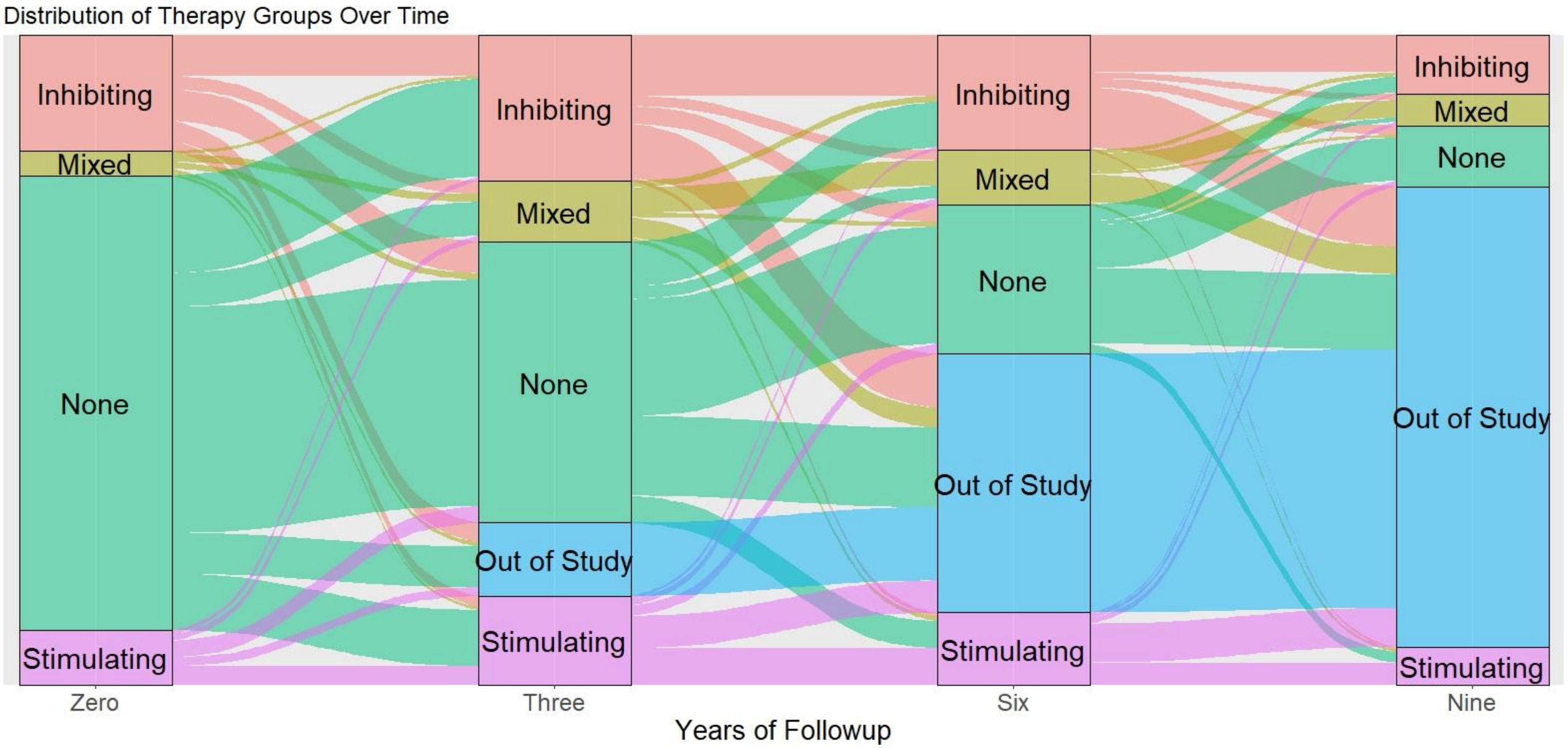

This figure shows transitions across time-dependent treatment groups over the study follow-up. Angiotensin II receptor type 2 and 4-stimulating antihypertensives defined as use of angiotensin II receptor blockers, dihydropyridine calcium channel blockers, and/or thiazides. Angiotensin II receptor type 2 and 4-inhibiting antihypertensives defined as use of angiotensin-converting enzyme inhibitors, ß-blockers, and/or non-dihydropyridine calcium channel blockers. Mixed antihypertensive users defined as both angiotensin II receptor type 2 and 4-stimulating and -inhibiting antihypertensives. “Out of Study” is defined as an individual being censored.

© 2023 Marcum ZA et al. *JAMA Network Open.*

eFigure 3. Results of Sensitivity Analysis - Multivariable Cox Proportional Hazards Model for Primary (ADRD) and Secondary (Vascular Dementia) Outcomes

Variable	P-value	Hazard Ratio of ADRD (95% CI)	P-value	Hazard Ratio of Vascular Dementia (95% CI)
Stimulating vs. Inhibiting Treatment	<0.001	0.83 (95% CI 0.78, 0.88)	0.004	0.79 (95% CI 0.67, 0.93)
Mixed vs. Inhibiting Treatment	0.029	0.94 (95% CI 0.88, 0.99)	0.102	0.88 (95% CI 0.76, 1.02)
No Treatment vs. Inhibiting Treatment	<0.001	0.91 (95% CI 0.87, 0.96)	0.726	1.02 (95% CI 0.91, 1.15)
Age, per 1 year increase	<0.001	1.10 (95% CI 1.09, 1.10)	<0.001	1.07 (95% CI 1.06, 1.08)
Female vs Male	<0.001	0.92 (95% CI 0.88, 0.96)	0.090	0.91 (95% CI 0.81, 1.01)
Black vs Non-Hispanic White	<0.001	1.38 (95% CI 1.27, 1.50)	<0.001	1.59 (95% CI 1.30, 1.95)
Hispanic vs Non-Hispanic White	0.123	0.91 (95% CI 0.80, 1.03)	0.178	0.80 (95% CI 0.57, 1.11)
Other Race/ethnicity vs Non-Hispanic White	0.005	0.87 (95% CI 0.79, 0.96)	0.009	0.70 (95% CI 0.53, 0.91)
Low-Income Subsidy	<0.001	1.50 (95% CI 1.44, 1.57)	<0.001	1.48 (95% CI 1.32, 1.66)
Atrial Fibrillation	<0.001	1.11 (95% CI 1.06, 1.17)	0.007	1.18 (95% CI 1.05, 1.33)
Congestive Heart Failure	<0.001	1.39 (95% CI 1.33, 1.46)	<0.001	1.36 (95% CI 1.21, 1.52)
Chronic Kidney Disease	<0.001	1.37 (95% CI 1.31, 1.43)	<0.001	1.49 (95% CI 1.34, 1.66)
Depression	<0.001	1.88 (95% CI 1.80, 1.96)	<0.001	2.54 (95% CI 2.28, 2.82)
Diabetes	<0.001	1.10 (95% CI 1.05, 1.14)	0.029	1.12 (95% CI 1.01, 1.25)
Ischemic Heart Disease	<0.001	1.12 (95% CI 1.07, 1.17)	0.918	1.01 (95% CI 0.90, 1.13)
Stroke	<0.001	1.71 (95% CI 1.63, 1.78)	<0.001	3.21 (95% CI 2.90, 3.56)
Anticholingeric Use	<0.001	1.13 (95% CI 1.08, 1.19)	0.220	0.93 (95% CI 0.82, 1.05)
NSAID Use	0.597	0.99 (95% CI 0.94, 1.04)	0.403	0.95 (95% CI 0.83, 1.08)
Statin use	<0.001	0.89 (95% CI 0.85, 0.93)	0.022	0.87 (95% CI 0.78, 0.98)
Use of Other Antihypertensive Drugs	0.043	0.95 (95% CI 0.91, 1.00)	<0.001	0.75 (95% CI 0.65, 0.86)

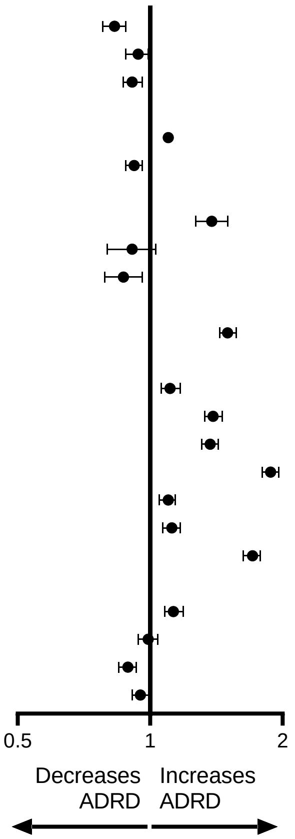

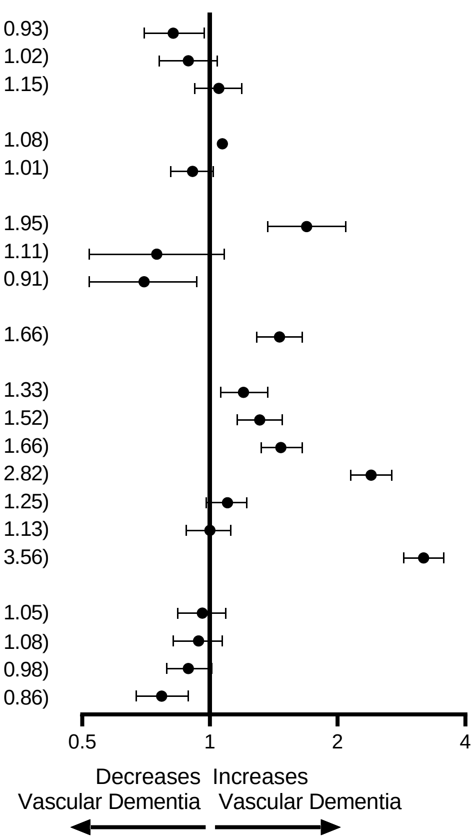

Abbreviations: ADRD, Alzheimer’s disease and related dementias; CI, confidence interval

This figure displays two separate multivariable Cox proportional hazards models for primary (ADRD) and secondary (vascular dementia) outcomes.

The stimulating group was defined as having at least 1 day of the 30-day interval with possession of stimulating antihypertensive medication and 0 days with possession of inhibiting medication. The inhibiting group was defined as having at least 1 day of the 30-day interval with possession of inhibiting antihypertensive medication and 0 days with possession of stimulating medication. The mixed group was defined as having at least 1 day of the 30-day interval with possession of inhibiting antihypertensive medication and at least 1 day with possession of stimulating antihypertensive medication. The non-users group was defined as having 0 days with possession of inhibiting antihypertensive medication and 0 days with possession of stimulating antihypertensive medication.

Angiotensin II receptor type 2 and 4-stimulating antihypertensives defined as use of angiotensin II receptor blockers, dihydropyridine calcium channel blockers, and/or thiazides. Angiotensin II receptor type 2 and 4-inhibiting antihypertensives defined as use of angiotensin-converting enzyme inhibitors, ß-blockers, and/or non-dihydropyridine calcium channel blockers. Mixed antihypertensive users defined as both angiotensin II receptor type 2 and 4-stimulating and -inhibiting antihypertensives.

© 2023 Marcum ZA et al. *JAMA Network Open.*
