# Supplementary material for: Association of New Use of Antihypertensives That Stimulate vs Inhibit Type 2 and 4 Angiotensin II Receptors With Dementia Among Medicare Beneficiaries
Source: JAMA Netw Open. 2023 Jan 4;6(1):e2249370. doi: 10.1001/jamanetworkopen.2022.49370 (PMC9856661; doi:10.1001/jamanetworkopen.2022.49370)
Supplement: Supplement 2. — Data Sharing Statement [file jamanetwopen-e2249370-s002.pdf]

Data Sharing Statement

Marcum. Association of New Use of Antihypertensives That Stimulate vs Inhibit Type 2 and 4 Angiotensin II Receptors With Dementia Among Medicare Beneficiaries. *JAMA Netw Open*. Published January 04, 2023. doi:10.1001/jamanetworkopen.2022.49370

Data
Data available: No

Additional Information
Explanation for why data not available: Data was obtained under a data user agreement that explicitly prohibits data sharing.
